# Distributed flux balance analysis simulations of serial biomass fermentation by two organisms

**DOI:** 10.1371/journal.pone.0227363

**Published:** 2020-01-16

**Authors:** Edward Vitkin, Amichai Gillis, Mark Polikovsky, Barak Bender, Alexander Golberg, Zohar Yakhini

**Affiliations:** 1 Department of Computer Science, Technion – Israel Institute of Technology, Haifa, Israel; 2 IBM Watson Health, Haifa, Israel; 3 Porter School of the Environment and Earth Sciences, Tel Aviv University, Tel Aviv, Israel; 4 School of Computer Science, The Interdisciplinary Center, Herzliya, Israel; University of Illinois at Urbana-Champaign, UNITED STATES

## Abstract

Intelligent biorefinery design that addresses both the composition of the biomass feedstock as well as fermentation microorganisms could benefit from dedicated tools for computational simulation and computer-assisted optimization. Here we present the BioLego Vn2.0 framework, based on Microsoft Azure Cloud, which supports large-scale simulations of biomass serial fermentation processes by two different organisms. BioLego enables the simultaneous analysis of multiple fermentation scenarios and the comparison of fermentation potential of multiple feedstock compositions. Thanks to the effective use of cloud computing it further allows resource intensive analysis and exploration of media and organism modifications. We use BioLego to obtain biological and validation results, including (1) exploratory search for the optimal utilization of corn biomasses—corn cobs, corn fiber and corn stover—in fermentation biorefineries; (2) analysis of the possible effects of changes in the composition of *K*. *alvarezi* biomass on the ethanol production yield in an anaerobic two-step process (*S*. *cerevisiae* followed by *E*. *coli*); (3) analysis of the impact, on the estimated ethanol production yield, of knocking out single organism reactions either in one or in both organisms in an anaerobic two-step fermentation process of *Ulva* sp. into ethanol (*S*. *cerevisiae* followed by *E*. *coli*); and (4) comparison of several experimentally measured ethanol fermentation rates with the predictions of BioLego.

## Introduction

Efficient and sustainable conversion of biomass into commerciable products, including food products, chemicals and fuels, currently is a major challenge for science, governments and industry across the globe [1]. Designing biorefineries requires addressing all aspects of the process, including biomass growth, harvesting and fermentation and also the distribution of products and handling waste [2]. The feedstock composition and its amenability to efficient fermentation by microorganisms are two important determinants of the efficiency of biorefineries [3]. While feedstock composition is constrained by local resources [4], there is much more freedom for designers in selecting fermentation configurations, including the constituent organisms.

Two major directions can be taken by fermentation designers. The first is to maximally utilize the biomass available at hand, when constrained by the given available feedstocks and when there is a need to identify potentially interesting products and design the corresponding configuration. The second is the efficient production of a certain molecule, when the desired product is known. In this case design aims to identify and adjust optimal feedstocks and their fermentation setup to achieve maximal production efficiency. Indeed, most biorefinery designers will tackle a mix of these challenges—a small set of feedstock candidates limited by local agricultural output and a small set of products with high commercial value to the local community (i.e. biofuels like ethanol, food supplements like carrageenan and more).

Starting with potential feedstocks available for fermentation (i.e. local species of corn or algae) and with the fermentation targets (i.e. bioethanol), designers of the fermentation process determine the constituent organisms for the process. The feedstock biomass is a composition of several types of compounds, such as amino acids (valine, histidine, lysine, etc.), monosaccharides (glucose, galactose, rhamnose, xylose, etc.) and polysaccharidic fibers (cellulose, hemicellulose, lignin, ulvan, etc.), fatty acids (myristic, oleic, palmitic, etc.) and others. Many of the domesticated, industrial organisms cannot metabolize some biomass components. Therefore, the selection of the fermentation configuration, to achieve maximum efficiency, can be challenging. Approaches to overcome this challenge include genetic modification of microorganisms with the goal to endow them with additional fermentation pathways, or, alternatively, to use several organisms [5,6], each specializing in fermenting different biomass components [7]. For example, some studies aim to genetically modify *S*. *cerevisiae*, which is first choice ethanol producer, to improve sugar (i.e. xylose) uptake mechanisms. Other studies aim to induce or to increase the required functionality in the organism by driving broader digestion rates, like in *E*. *coli* [8,9].

Fermentation processes utilizing bacterial communities require more diverse equipment, expertise in cultivating several organisms, and understanding of inter-organism interactions and of how they compete for resources [10]. Mathematical modelling of community-based fermentation processes is also more complicated, since the natural inter-organism interactions are still not-sufficiently understood [11] and as mapping metabolites between models of different organisms is technically complicated, mostly due to historical inconsistencies of naming conventions [12,13]. The OptCom [11] methodology proposes a computational framework for modelling inter-species interactions, aiming to balance individual vs. community level fitness criteria. Similarly, cFBA [14] integrates inter-species interactions and performs modelling that maximizes the growth rate of entire bacterial communities.

Serial fermentation is a design approach that offers tighter and more predictable control of inter-organism relations and reactions, thus enabling better process optimization [15–18]. Briefly, in this process each organism is grown separately. Residual media together with decomposed biomass, resulting from this first step, are then transferred to the next organism, serially. One particularly interesting subclass of serial fermentation schemes is a two-step fermentation process, in which only two organisms participate in the fermentation.

In previous work we introduced BioLego—a platform that simulates the efficiency of biomass fermentation in two-step processes [15,16,19]. This current work leverages Microsoft Azure Cloud to enhance the BioLego framework and to allow high-scale simulations, exploring a large multiplicity of scenarios, of the efficiency of biomass fermentation in two-step processes. Tasks addressable by the cloud-based approach include simultaneously analyzing multiple fermentation configurations and comparing the potential of several feedstock biomasses. Most importantly—the cloud-based approach allows for analyzing the effects of different media compositions and of genetic modifications of the organisms, a task that is highly resource-intensive.

Our biological and validation results include (1) exploratory search for optimal biomass utilization setup for three different types of corn biomasses—*corn cobs*, *corn fiber* and *corn stover*; (2) analysis of possible effects of changes in the composition of *K*. *alvarezi* algal biomass on the ethanol production yield in the anaerobic two-step process (*S*. *cerevisiae* followed by *E*. *coli*); (3) analysis of impact on the estimated ethanol production yield of knocking out single organism reactions either in one or in both organisms in anaerobic two-step process that ferments *Ulva sp*. into ethanol (*S*. *cerevisiae* followed by *E*. *coli*); and (4) comparison of experimental measurements of ethanol fermentation efficiency with the efficiency predicted by BioLego system.

Our results, as described in the current paper, significantly impact several aspects of biorefinery design processes. The BioLego code now has fully functional important features and capabilities, including support for performing knock-out analysis on a large scale, as well as components that connect it to cloud services. Such components are essential for performing large-scale tasks in reasonable time. In addition, our biological results validate our modelling approach for serial processes and shine light on the potential of using knockouts to increase ethanol production in such systems. Finally, our specific findings, as related to corn and to *Ulva sp*. may be useful in the context of actual production.

## Materials and methods

This section briefly covers all implementation details of BioLego 2.0 system. All the source code, detailed explanations, installation instructions, simulation results and Supplementary materials are available at http://wassist.cs.technion.ac.il/~edwardv/STORAGE/biolego2_data/

### 2.1. Serial biomass fermentation by two organisms

Incomplete understanding of interactions between organisms and differences in nomenclatures are two major challenges in modeling community-based fermentation processes. Leveraging the nature of processes composed of separate steps, we address these problems by a flexible modular modelling approach. Specifically—our models encapsulate existing metabolic models in dedicated envelopes that can then be combined using defined interfaces. This software design approach thus produces different fermentation configurations by combining models with each other using interface that is metaphorically similar to the interface between LEGO bricks.

For completeness we briefly describe the approach herein. Full details are available at [16].

#### 2.1.1. Flux Balance Analysis

Flux Balance Analysis (FBA) framework is a basis for BioLego mathematical simulations of biomass utilization and ethanol production yields. FBA is a sub-class of Constraint-Based Modeling (CBM) mathematical modeling approaches. CBM analyzes internal reaction fluxes based solely on simple physical-chemical constraints, such as reaction stoichiometry and metabolic flux constraints, without requiring exact enzyme kinetic data. CBM approach enables the prediction of organism growth rates based only on reaction stoichiometry and directionality. FBA-based approaches have a wide range of applications including phenotype analysis, bioengineering and metabolic model reconstructions [20–22].

The reaction stoichiometry in a metabolic model is represented by stoichiometric matrix *S*, wherein *S*_*m*,*r*_ corresponds to stoichiometric coefficient of metabolite *m* in the reaction *r*. The vector of metabolic fluxes that are carried by the model reactions, normally denoted as $\vec{v}$, is constrained both by mass-balance (Eq 1) and by maximal/minimal feasible fluxes $\vec{v^{UB}}$ and $\vec{v^{LB}}$ (Eq 2).

$$S*\vec{v}=0$$ (1)

$$\vec{v^{LB}}\leq \vec{v}\leq \vec{v^{UB}}$$ (2)

Although $v_{r}^{UB}$ and $v_{r}^{LB}$ are set to ±infinity for many reactions due to lack of knowledge, the solution space is not actually unbounded; it is always constrained by the feedstock media uptake rate. In our specific case, the actual media uptake rate is less important, as we are interested in the total conversion (in %) of biomass into ethanol (or other target product), rather than in specific reaction rates. We therefore assume a media uptake rate of 1gDW*gDW^-1^*h^-1^ (1 gram of media dry weight per 1 gram of bacteria dry weight per hour) of media and enforce it through the media transporter reactions. We then calculate the yield of biomass-to-ethanol conversion accordingly. Note, that this uptake rate is very similar to the commonly accepted rate in *E*. *coli*. For example, the glucose uptake rate commonly assumed for *E*. *coli* is 1.8gDW*gDW^-1^*h^-1^ (which according to mole-to-gram transformation formula (*Amount*[*mol*] = *Weight*[*g*] ⁄ *Molecular*_*Weight*[*g* * *mol*^−1^]) corresponds to 10mmol*gDW^-1^*h^-1^ (using glucose molecular weight of 180g*mol^-1^) of glucose uptake rate defined in iJO1366 [23]), when grown on glucose minimal media [23].

FBA, a special case of the CBM framework, assumes that the metabolic network of the studied organism is regulated (e.g. by evolutionary processes) to maximize some cellular function, which is usually an organism growth rate for unicellular organisms [24]. The FBA formulation is summarized in Eq 3:

$$\begin{array}{c} BM_{MAX}=\underset{\vec{v}}{\text{max}}\left\{v_{Growth}\right\}\\ \text{s}.\text{t}.:\left[\begin{array}{l} \begin{array}{l} S*\vec{v}=0\\ \vec{v^{LB}}\leq \vec{v}\leq \vec{v^{UB}} \end{array}\\ \begin{array}{c} \sum v_{media\;transporters}=1\frac{gDW\left(media\right)}{h} \end{array} \end{array}\right] \end{array}$$ (3)

Here, *v*_*Growth*_ is an artificial growth reaction, representing the organism’s growth rate. This quantity converts all the organism cellular components into a single output variable representing a unit of biomass; *v*_*media transporters*_ is a generic name for all transporter reactions and *BM*_*MAX*_ is an estimated maximal organism growth rate under the given constraints. Optimization process may identify many possible sets of fluxes that both maximize *v*_*Growth*_ and satisfy all the CBM constraints. That is—the optimum is often attained by a large set of possible solution vectors, $\vec{v}$.

We are often interested in finding ranges of certain components of the vector $\vec{v}$, when taken from the set of values that attain the optimal value as above. Note reactions other than *v*_*Growth*_ (in particular, ethanol production) will potentially have a range of possible values within (affine) subspaces of fluxes that maximize growth rate (Eq 3). Modelling, known as Flux Variability Analysis (FVA)[25], aims to estimate this range. That is—FVA aims to evaluate both the maximum (Eq 4a) and the minimum (Eq 4b) possible values for the target reaction *v*_*Target*_, amongst the fluxes that attain maximum growth:

$$\begin{array}{c} a)\;\underset{\vec{v}}{\text{max}}\left\{v_{Target}\right\}\;b)\;\underset{\vec{v}}{\text{min}}\left\{v_{Target}\right\}\\ \text{s}.\text{t}.:\left[\begin{array}{l} v_{Growth}^{LB}=BM_{MAX}:\text{Solution}\;\text{of}\;\text{Equation}\;3\\ S*\vec{v}=0\\ \vec{v^{LB}}\leq \vec{v}\leq \vec{v^{UB}}\\ \sum v_{media\;transporters}=1\frac{gDW\left(media\right)}{h} \end{array}\right] \end{array}$$ (4)

The deletion of certain reactions, also referred to as *reaction knockouts*, may have a major impact on the distribution of the model fluxes. Such deletions are enabled through genetic modifications, mostly gene knock outs or knock downs [26]. FBA provides means for analyzing this impact by nullifying reaction boundaries of the deleted reaction. However, screening of all possible organism knockouts is a heavy computational task, since common metabolic models consist of thousands of reactions. Therefore, such screening is usually replaced by a search for the solution maximizing the production yield [27,28].

In BioLego we use this general framework to perform evaluation steps and then combine them, as further described below.

#### 2.1.2. Modular approach of BioLego

**Fig 1:**
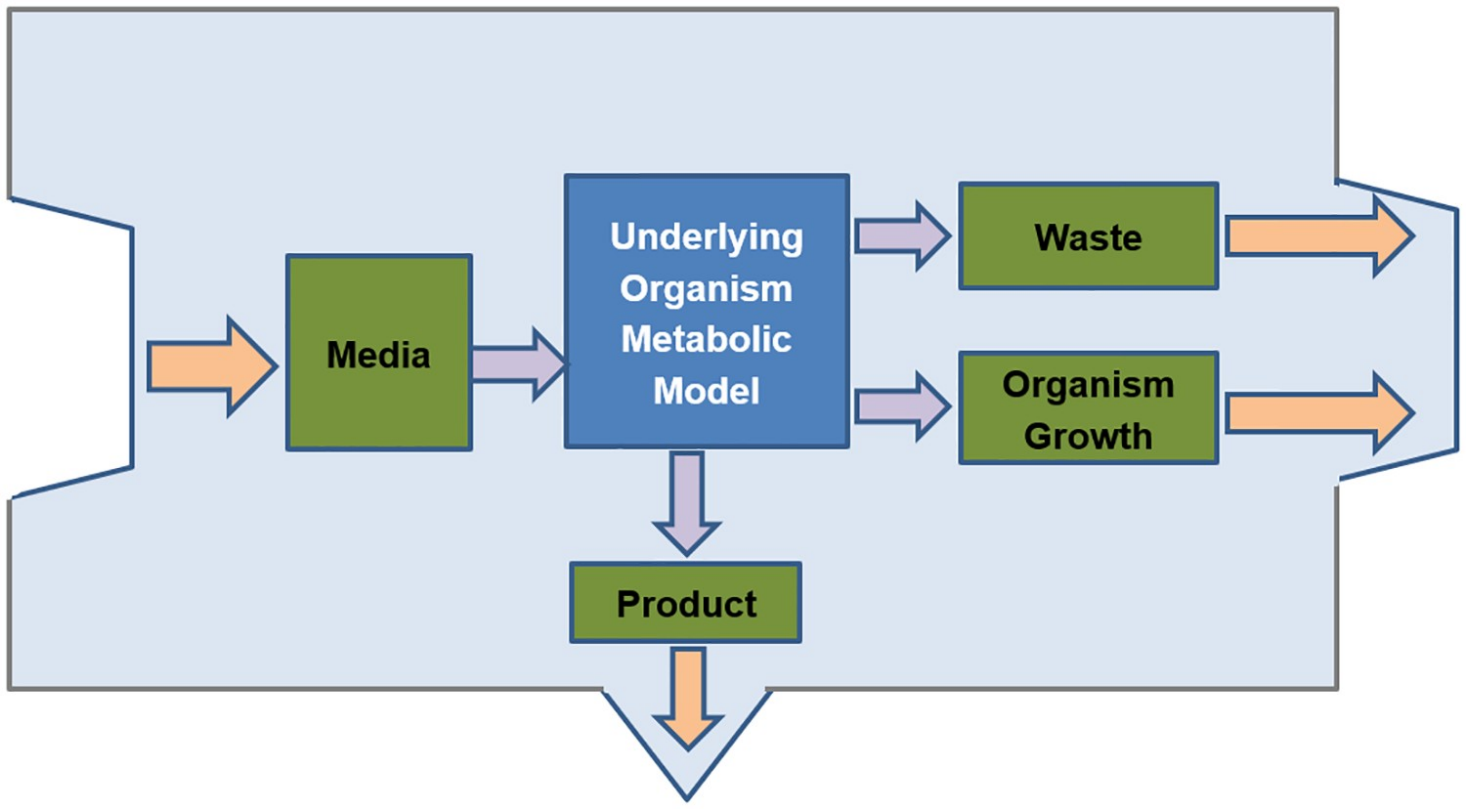
Single-module for existing organism metabolic model. This module is composed of five chambers: (i) *Media*—representing received biomass feedstock media; (ii) *Internal*—consisting of the known metabolic model of the organism; (iii) *Growth*—consisting organism cellular components; (iv) *Product*—representing desired target metabolite; and (v) *Waste*—representing all non-digested media residuals and molecules by organism as growth by-product.

To evaluate the expected yield of the multi-organism fermentation we combine the metabolic models of different participating organisms and simulate them together. Specifically, we encapsulate literature organism metabolic models in a dedicated envelope layer (Fig 1). This allows overcoming nomenclature differences and enforcing inter-organism interactions only to a desired predefined set. Practically the latter makes sense, since in serial fermentation all the constituents moving from one organism to the following is under our control.

For each organism we create a single module, composed of two principle parts—the *Internal* chamber, which is essentially an existing organism metabolic model, and the four chambers of the envelope layer: *Media*, *Growth*, *Waste* and *Product* (Fig 1). *Media* refers to the set of media compounds received by organism; *Growth* refers to the set of molecules comprising the organism biomass; *Product* refers to the desired product and *Waste* refers to all the compounds either non-digested from media or non-product molecules extracted to the outside as a side-effect of metabolic processes. Obviously, each such module should be constructed dynamically according to the setup-specific media composition and the defined target product molecule. We enforce the inter-chamber metabolic fluxes to be unidirectional. In addition, no direct metabolic flux between the internal chamber and the external environment is allowed, which results in full control over all inputs and outputs of the system.

The exact formulation of this envelope, as well as of the equations for modeling serial combinations of organism modules into a two-step fermentation pipeline, was previously described [16,29].

### 2.2. Implementation of Azure-based distributed system

Simulating each scenario of interest can take a few seconds. Working on a single machine becomes infeasible in cases when the number of such scenarios is in the millions (from example when simulating several knockouts in one organism or simultaneous knockouts in different organisms). We address this challenge by developing a flexible and scalable system leveraging the MS Azure Cloud capabilities. This particular system is just an example of a cloud computing environment that can be used in this context. S1 Fig presents the simplified snapshot of the resulted distributed architecture. The entire system’s complexity is transparent to the end user. Once the system is set up, no specific programming skills, such as Matlab knowledge, are required. The user provides the input in HTML and/or XLS (i.e. CSV) format and, after the simulation terminates, receives the results in HTML and CSV formats.

In the background we first place a request on the entrance server, which can run either locally (as in our implementation) or remotely. It verifies the request correctness and generates setup-specific requests per each desired fermentation configuration (selected pipe of organisms, media and production target) to Azure Cloud side via dedicated request submission queue (*setup-requestq*). This stage is parallelized into several instances on Azure Cloud side to support simultaneous construction of multiple fermentation setups. The resulting model, describing requested fermentation setup, is placed in a dedicated blob (file storage entity) in the Azure Cloud and the local server is notified by dedicated notification queue (*setup-resultq*).

Upon receiving such notification, the local server generates a set of tasks specific to each fermentation setup, which may involve slight modifications of the resulted model. Specifically, it may include an update in reaction boundaries for several model reactions, thus giving means to perform media gradient and sensitivity analyses; to perform estimation within same setup of various oxygen amounts; and to simulate sets of reaction knockouts. The generated set of single tasks is sent to Azure Cloud side for the evaluation via a dedicated queue (*task-requestq*). This stage is also parallelized into several instances to support tasks multiplicity. The details of estimation results (i.e. flux values of specific reactions) are placed in a dedicated result blob and the local server is notified with result summary via dedicated notification queue (*task-resultq*).

To address computationally challenging estimation problems, such as reaction knockout analysis, the BioLego architecture on the local server side includes few additional components dedicated to handling large-scale tasks. Specifically, we parallelize both the sending of task calculation requests and the processing of the received single-task results.

The entire simulation framework is implemented in Perl for management of model and media files, in Matlab for FBA formulations and in GNU Linear Programming Kit [30] for solving the resulting linear programming problems.

### 2.3. Media sensitivity analysis

Sensitivity analysis aims to understand the sensitivity of estimated fermentation efficiencies to the presence of the certain components in the received media. Specifically, we are interested in the relative change (in %) in the production yield, further on *relative yield*. To this end, we iteratively remove media components one at a time by nullifying the value of the appropriate reaction boundary ($v_{r}^{UB}$) in *Media* chamber (Eq 2 and Fig 1) and repeating the estimation process. The resulting relative yield is a ratio of the new and the original (both minimal and maximal) production yields (Eq 5).

$$\text{RelativeYield}\left(compound\right)=100\%*\frac{v_{TotalProduct}\left\{s.t.:v_{Outside\to Media\left(compound\right)}=0\right\}}{v_{TotalProduct}\left\{original\;setup\right\}}$$ (5)

### 2.4. Optimization of biomass composition by media gradient

In addition to elimination of certain media compound (as in 2.3), biorefinery designer can be interested in the minor media modifications, for example resulted from different feedstock crop growth conditions. Media content optimization by gradient analysis is targeted to understand the sensitivity of estimated fermentation efficiency to minor changes in media. To this end, we iteratively increase the amount of media components one at a time by adding small *δ* (*δ* = 1mg/gDW of media) value to appropriate reaction boundary ($v_{r}^{UB}$) in *Media* chamber (Eq 2 and Fig 1) and repeating the estimation process. The resulting gradient component is equal to the difference between new and the original (both minimal and maximal) production yields (Eq 6).

Gradient(compound)=[vTotalProduct{s.t.:vOutside→Media(compound)+δ}−vTotalProduct{originalsetup}]δ (6)

### 2.5. Fermentation pipeline optimization by reaction knock-outs

Biomass fermentation pipeline can be optimized not only by a smart selection of participating organisms and their ordering, but also by internal organism modifications. Our software supports the evaluation of the effect of deleting any selected subset of reactions on the resulting production rates. Mathematically, to simulate the reactions’ knockouts we nullify the values of boundaries of corresponding reactions ($v_{r}^{UB}=v_{r}^{LB}=0$) and repeat the estimation process [27,28].

In the BioLego system we provide an option to simulate any number of simultaneously deleted reactions in each organism in the pipe. Specifically, the user has an option to select in each organism a specific reaction to knockout or to perform an exhaustive scanning of all possible reactions and reaction combinations. This selection can be performed in a nested scheme, whereby at each repetition the new knockouts are added to the previously selected ones.

### 2.6. Experimental two-step fermentation with *S*. *cerevisiae* and *E*. *coli*

#### 2.6.1 Microbial cultivation

A fresh culture of *Saccharomyces cerevisiae* (Ethanol Red, Batch 62186/2, ‘Leaf’, France) or *Escherichia coli*, strain K-12 MG1655 wild type (WT) were prepared by smearing the microorganism from the glycerol stock (in -80 °C) on agar plates with rich media. For *S*. *cerevisiae* the rich medium was yeast extract peptone dextrose (YPD). YPD medium was composed of 1 L of distilled water (Zalion, Israel), 10 g/L yeast extract (BD, Bacto^™^ Yeast Extract), 20 gr/L peptone (BD, Bacto^™^ peptone) and 20 g/L glucose (Merck, D(+)-glucose [31]). For *E*. *coli* the rich medium was Lysogeny broth (LB) agar plates. The LB medium composition was 1 L of distilled water, 10 g/L NaCl (Bio-Lab, Israel), 10 gr/L tryptone (Neogen, U.S.A.) and 5 g/L yeast extract (BD, Bacto^™^ Yeast Extract [32]). In order to prepare YPD or LB solid medium, an agar (Merck, Agar-agar) 15 g/L was added to the medium. After autoclaving in 121 °C for 30 minutes (Tuttnauer 2540MLV, 186 Netherlands), the liquid cooled and poured into 90mm petri dishes. After the plating, the microorganisms’ plates were incubated in 32 °C or 37 °C for *S*. *cerevisiae* and *E*. *coli*, respectively.

#### 2.6.2 Ulva biomass hydrolysis

The biomass of *Ulva lactuca* was cultivated offshore, detailed cultivation process and cultivation conditions described in [33]. After harvesting the biomass, it was dried in 40 °C up to achieve a constant weight. Then, the dry biomass is milled with an electric grinder (Grinding machine, Henan Gelgoog Machinery GG9FZ-19) until powdered. For obtaining a constant particles size (0.063–0.125 mm) the powder passed through a size-selective metal-mesh (Sieve Sets S3076, Aquatic Eco-systems). The *Ulva* powder was then hydrolyzed thermochemically and, next, the residual was also hydrolyzed enzymatically.

The thermochemical hydrolysis was performed by autoclaving in 121°C for 30 min in 50 mL autoclavable tubes (Nalgene^™^ Oak Ridge High-Speed PPCO 185 Centrifuge Tubes, Thermo-Fisher Scientific, CA) with 2 g of the *Ulva* powder and with 20 mL of 2% sulfuric acid (v:v) (Sigma-Aldrich, Israel). After the autoclaving, the hydrolysate neutralized to pH 6 with NaOH (Merck, Sodium hydroxide), with about 1.7 ml of 3M NaOH. Additionally, for stabilizing the hydrolysate’s pH a 3.3 ml of 0.5M phosphate buffer was added (Phosphate Buffer Powder, Sigma-Aldrich, Israel) [34]. At the end of thermochemical hydrolysis, the solid and the liquid were separated using centrifuge (Rotanta 46 RSC, Hettich, Germany) at 4000 rpm for 7 minutes. The liquid phase was transferred to a sterile 50 mL test tube (Miniplast, Israel) while the solid phase left in the 50 mL autoclavable tubes went to further enzymatic hydrolysis.

In order to enzymatically hydrolyze the solid phase, 20 mL of sodium acetate buffer (200 μM) (Sigma-Aldrich, Israel) were added to the 50 mL autoclavable tubes with the solid phase. Additionally, a mixture of enzymes was added with the following active units (in 20 ml): amyloglucosidase 36 U, α-amylase 19 U and cellulase 33 U. The enzymatic hydrolysis was done horizontally in an orbital shaker incubator (Incu-Shaker Mini, Benchmark, USA) for 24 h at 45 °C with 150 rpm [35,36].

Finally, 25 ml of the thermochemical hydrolysate and 20 ml of the enzymatic hydrolysate were mixed and the liquid phase was separated using a centrifuge, at 4000 rpm for 7 minutes. This liquid phase was used for the two-step fermentation.

#### 2.6.3 Two-step fermentation of Ulva hydrolysate

The *Ulva* hydrolysate was fermented in a two-step fermentation process, after adding a microbial starter. The microbial starters were prepared by transferring a single colony of each microorganism (*S*. *cerevisiae* or *E*. *coli*) from rich solid media (YPD or LB) to 15 mL sterile test tubes (Culture tubes, PP, 2-stage-cap, Bar-Naor, Israel) with 2 mL of hydrolysate. The microorganisms incubated in the hydrolysate during an overnight at 32 °C for *S*. *cerevisiae* and 37 °C for *E*. *coli*, both shaken in an orbital shaker incubator at 150 rpm.

The two-step fermentation was applied in four different microbial sequential combinations as described in S4 Table. Every fermentation started after adding 75 μL of starter to the hydrolysate. The microbial concentrations in the starters were: *S*. *cerevisiae* 0.43 OD 600_nm_ and *E*. *coli* 0.33 OD 600_nm_ (Tecan Infinite 200 PRO, TECAN, Switzerland). First fermentations took place in 3 mL of *Ulva* hydrolysate in sealed 10 ml autoclavable tubes (Nalgene^™^ Oak Ridge High-Speed PPCO 185 Centrifuge Tubes, Thermo-Fisher Scientific, CA). The microorganisms in both fermentation steps were incubated for 24 h, in orbital shaker incubators with similar temperature and shaking conditions as used for the starters. After every first fermentation step, the ethanol was removed from the samples and the microorganisms were deactivated. The ethanol was evaporated from the samples by heating to 80 °C for 25 min in a water bath. The deactivation of the microorganisms was done in an autoclave, 121°C for 30min. At the end of every fermentation, the test tubes were weighed, and samples were taken for analyzing monosaccharides and the ethanol content.

#### 2.6.4 Sugars and ethanol measurements

The sugars were identified and separated with HPIC (Dionex ICS-5000, Thermo Fischer Scientific, CA, USA), with the same chemicals, separation method, analytical column, electrode and detector as used in [34]. Prior to the HPIC analysis, the samples were diluted 50 times and were filtered with 22 μm syringe-filter (Millipore, USA) into the HPIC vials. The sugars in the samples were quantified after comparing them to reference standards of rhamnose, galactose, glucose, xylose, fructose and glucuronic acid (chemicals for standards from Sigma-Aldrich, Israel).

The ethanol was measured with an ethanol assay kit (K-ETOH, Magazyme, Ireland) using a spectrophotometer (Tecan, infinite M200 PRO) at OD 340_nm_.

## Results

This section briefly covers experiments performed for this study. This includes (1) exploratory search for optimal biomass utilization setup for three different types of corn biomasses; (2) analysis of possible effects of changes in composition of *K*. *alvarezi* algal biomass on the ethanol production yield in the anaerobic two-step process; (3) analysis of the impact of knocking out single reactions either in one or in both organisms on the estimated ethanol production yields, under an anaerobic two-step fermentation process of *Ulva sp*. into ethanol; and (4) evaluation of BioLego predictions using experimentally measured data (see Materials and methods for details of the experimental measurement).

### 3.1. Optimizing corn utilization

We performed an exploratory search for optimal biomass utilization for three distinct types of corn biomasses—corn cobs, corn fiber and corn stover. The exact biomass compositions for this experiment were derived from [37–39]. Five potential fermentation targets were evaluated: ethanol, acetone, 1-butanol, (R)-propane-1,2-diol and (S)-propane-1,2-diol.

For each tested biomass and fermentation target we performed simulations for both single-step and for two-step fermentations under either aerobic or anaerobic conditions for four organism models (*E*.*coli* based on model iJO1366[23], *C*. *acetobutylicum* based on model iCAC490[40] and two models of *S*. *cerevisiae* based on Yeast5[41]—with and without xylose digestion mechanism) currently integrated in BioLego flow. In total, this experiment included 480 different simulations. Azure cloud side for this experiment included 10 instances of model setup constructors each with 2 threads running on Standard A1v2 nodes with 1 core and 2048MB memory and 10 instances of single fermentation task evaluation instances, running in 3 threads on Standard A1v2 nodes with 1 core and 2048MB memory. The calculation of all scenarios, which included construction of 240 model configurations and 480 single fermentation task simulations, was completed in 21 mins (estimated single-thread runtime is ~1.5–2 days).

Our results are provided in S1 Table. For ethanol production we predict corn cobs to be most promising among the studied corn media compositions. Maximal production yields (for all media compositions) were predicted for anaerobic two-step scenarios starting with *S*. *cerevisiae*. For (S)-propane-1,2-diol production we predict corn fibers to be most promising among the studied corn media compositions. Maximal production yields (for all media compositions) were predicted for two-step scenarios starting either with *S*. *cerevisiae* or with *C*. *acetobutylicum*. Interestingly, all the scenarios with positive production yields (including the aerobic one) include *E*. *coli* as one of the fermenting organisms.

### 3.2. Sensitivity and gradient analyses for ethanol production using fermentation of *Kappaphycus alvarezzi* biomass

We analyzed the possible effects of changes in composition of *K*. *alvarezi* algal biomass on the ethanol production yield in the anaerobic two-step process, in which first organism is *S*. *cerevisiae* and the second one is *E*. *coli*. As was shown previously, this fermentation configuration is predicted to result in high ethanol production [42].

*K*. *alvarezi* media is composed of 37 different compounds, testing each requires single fermentation estimation task. For this experiment Azure Cloud side included 5 instances of single fermentation task estimating instances, running in 3 threads on Standard A1v2 nodes with 1 core and 2048MB memory. Both sensitivity analysis (Eq 5) and gradient estimations (Eq 6) finished after 4 minutes (estimated single-thread runtime is ~3–4 hours).

Our results for media sensitivity analysis are available in S2 Table. Naturally, the ethanol production yields appeared to be most sensitive to the presence of galactose and glucose in media. Omission of these metabolites respectively decreased the ethanol production yields to 46–53% and 44–57% (range is minimal to maximal predicted productions). Surprisingly, omission of some media components (glutamic acid, glycine, tyrosine and leucine) slightly (up to 0.3%) increased predicted maximal ethanol production. These findings can be explained by decreased organism growth rate resulted from the component omission, which in turn led to increased amounts of other media components available for ethanol production.

Our media gradient analysis results are available in S2 Table. Interestingly, gradient estimation results for minimal and maximal ethanol production yields differ from each other. When considering the minimal ethanol production rate, we observe the highest gradient for various monosaccharides media components—mannose, glucose, galactose, xylose, arabinose; and lowest for glutamic acid, aspartic acid and asparagine. When considering the maximal ethanol production rate, we observe higher values not only for sugars but also for some amino acids, like threonine (highest) and serine. Interestingly, asparagine, aspartic acid and serine have positive directional derivative values for maximal rates and negative directional derivative values for minimal predicted ethanol production rates.

### 3.3. Two-step fermentation with knock-outs in each organism

We analyzed the anaerobic two-step fermentation process of *U*. *lactuca* into ethanol, in which first organism is *S*. *cerevisiae* and the second one is *E*. *coli*. Here we investigated the impact of knocking out single organism reaction either in one or in both organisms on estimated ethanol production yield.

The *S*. *cerevisiae* model is composed of 2,280 metabolic reactions, and the *E*. *coli* model is composed of 2,914 metabolic reactions. In total, this leads to 6,649,115 possible single knockout scenarios. To handle a task of this scale, the local server side was configured to operate 25 task calculation-request-sending threads and 10 result-processing threads. The Azure Cloud side for this calculation included 45 single fermentation task estimating instances, each one running with 10 threads on Standard D3v2 nodes with 4 cores and 14336MB memory. Running time for this experiment was 126 hours (estimated single-thread runtime is ~60–65 years). Notably, this time can be significantly reduced (up to 50–70%), with the same Azure Cloud side configuration, by using a more powerful local server machine.

**Fig 2:**
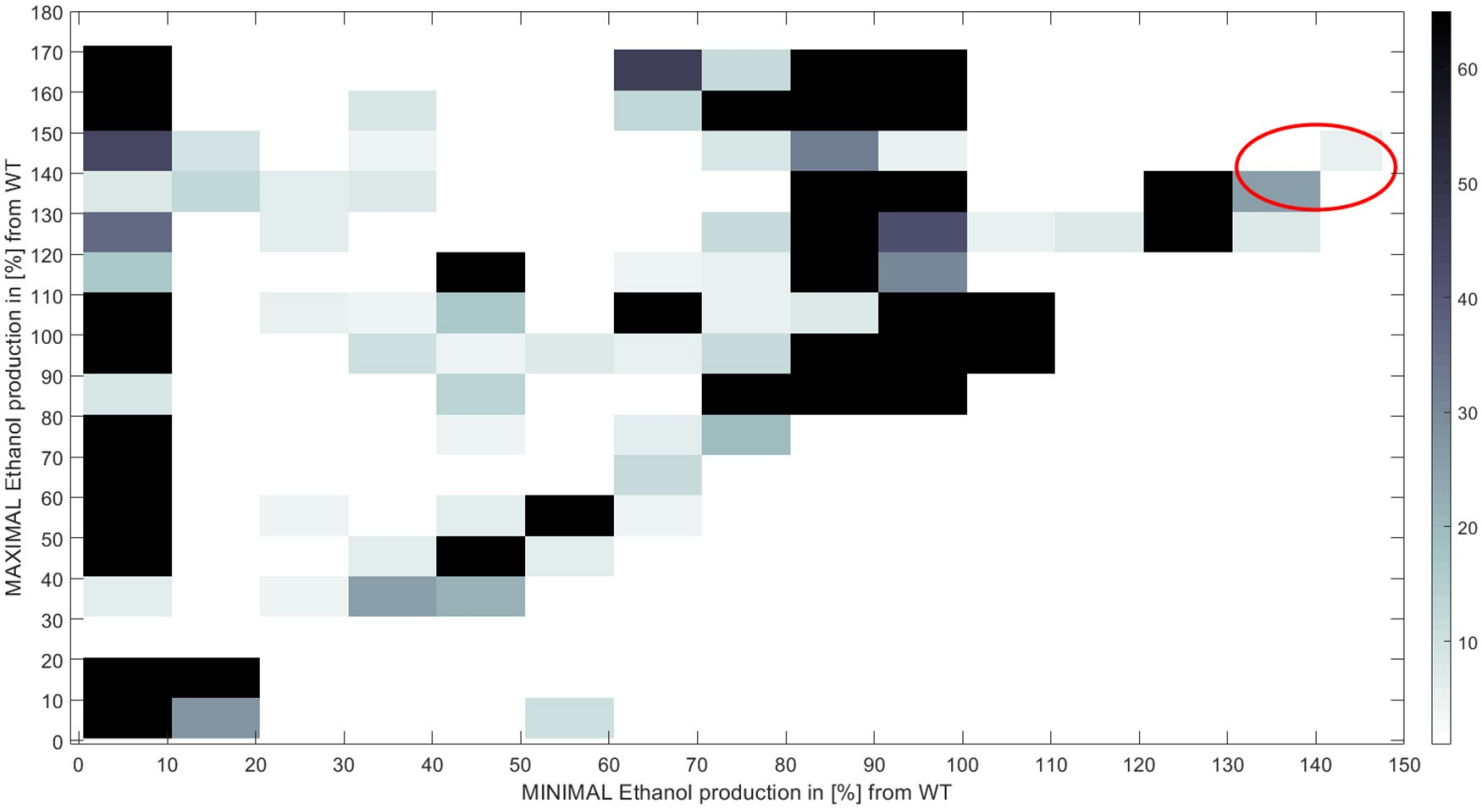
Heatmap of the expected minimal and maximal ethanol production yields per knocked pair of reactions, in [%] of wild type (WT) respectively minimal and maximal ethanol production yields. The red circle marks 24 reaction pairs with both minimal and maximal ethanol productions above 130%.

The results of the analysis results are described in S3 Table. Histograms of the predicted impact of all knockout pairs (one reaction in *S*. *cerevisiae* and one in *E*. *coli*) on both minimal and maximal ethanol production rates are presented in S3 Table and in S2 Fig. The results describing the predicted impact of all knockouts on both minimal and maximal ethanol production rates are presented as a heatmap in Fig 2. Minimal ethanol production yield is predicted to be increased to at most 146% of the wild type (WT) yields (for effectively one knockout candidate pair); while for maximal ethanol production this number reaches 170% of WT (for 867 knockout candidate pairs). Interestingly, note that the below-diagonal section of the heatmap is almost empty, meaning that most of the knockout pairs either equally affect both minimal and maximal ethanol production rates (pairs on the diagonal) or have a greater effect on the maximal ethanol production (pairs above the diagonal).

There are 24 reaction pairs for which knockouts are predicted to increase both minimal and maximal ethanol productions by more than 130%. These reaction pairs are combination of 2 reactions from *E*. *coli* and 12 reactions from *S*. *cerevisiae*. The *E*. *coli* reactions consist, in fact, of one artificial and one real reaction working in a linear process of proton (H+) extractor. This means that in effect this result represents a single unique reaction in *E*. *coli*. This reaction pairs with 9 non-orphan *S*. *cerevisiae* reactions, leading to 9 interesting combinations that can potentially be further explored. The *E*. *coli* reaction can be encoded by one of the following *E*. *coli* genes: b0241, b0929, b1377, b2215. The 9 *S*. *cerevisiae* non-orphan reactions map to 35 known genes. This process can be encoded by one b0241, b0929, b1377, b2215 genes. The *S*. *cerevisiae* side includes 9 reactions with overall 35 known related genes. Analyzing these genes with GOrilla [43,44] resulted mostly in proton-transporting and biosynthesis processes (p-value of 1E-15 and less, S3 Fig).

### 3.4. Experimental validation of prediction quality

We compared the experimentally measured ethanol fermentation efficiency with the efficiency predicted by BioLego system in four different scenarios: two serial two-step fermentation scenarios (*S*. *cerevisiae* followed by *E*. *coli* and *E*. *coli* followed by *S*. *cerevisiae*) and 2 single step fermentations (*S*. *cerevisiae* and *E*. *coli*) with presence of small initial amount of oxygen. Specifically, organisms were grown for 24 hours in closed vials with small amount of air. In these experiments we measured ethanol production yield as a function of initial amount of sugars (rhamnose, galactose, glucose, xylose, fructose and glucuronic acid) in *U*. *lactuca* biomass. The experimental details are provided in Section 2.6 and S4 Table.

**Fig 3:**
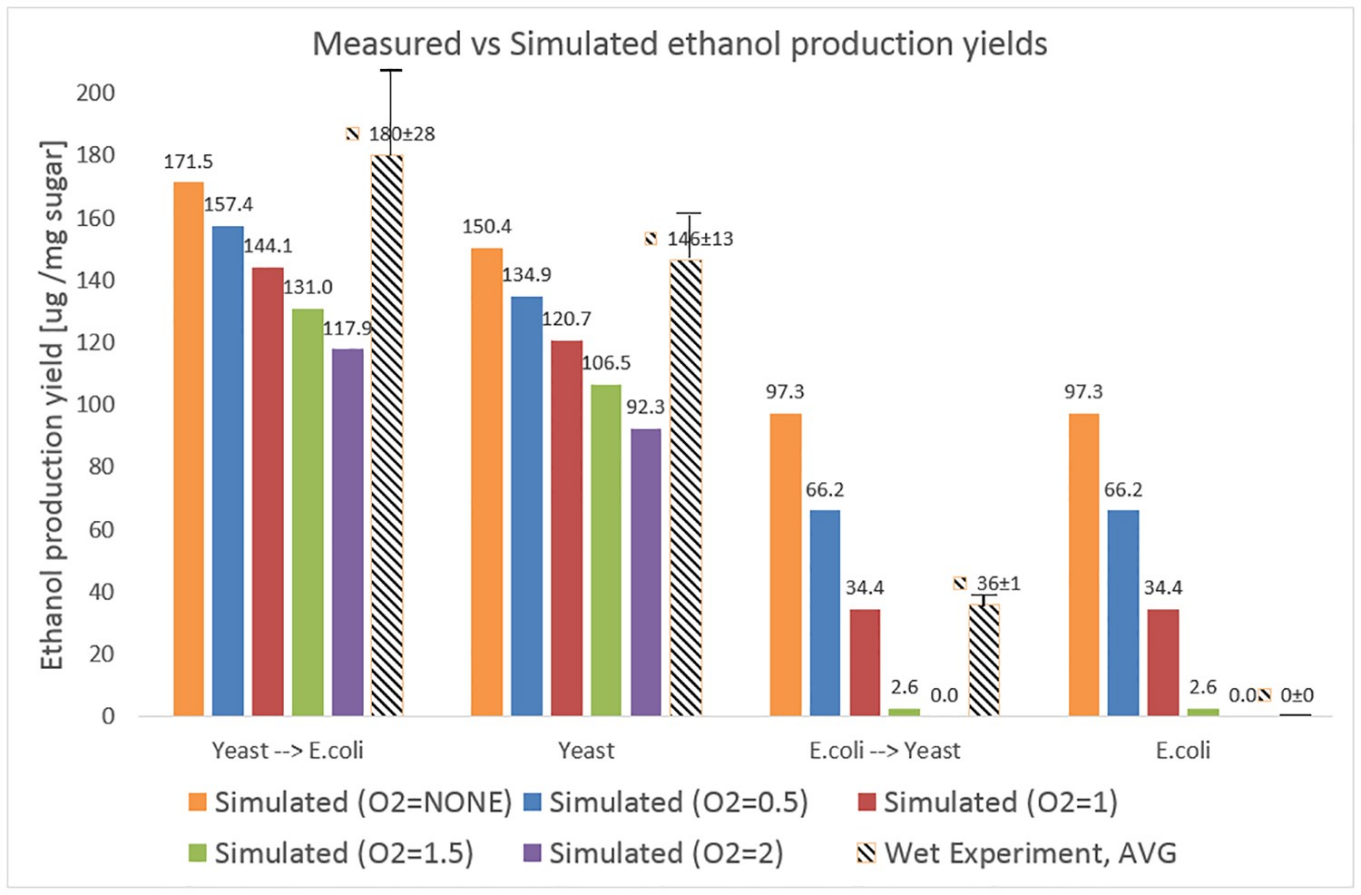
Experimental validation of BioLego predictions. Values for wet lab experiment are displayed ±STD.

During BioLego simulations (same computational setup as in Section 3.1) to match the experimental conditions, we assumed that *U*. *lactuca* biomass is composed purely from the measured sugars (rhamnose, galactose, glucose, xylose, fructose and glucuronic acid) and evaluated ethanol yields under a small allowed influx of oxygen (0-2mmol*gDW^-1^*h^-1^). Fig 3 demonstrates the similarity of the predicted maximal ethanol results with the performed measurements.

Both scenarios which started with *S*. *cerevisiae* demonstrate good alignment (increasing with the decrease of oxygen influx) with actual measurements. In both scenarios started with *E*. *coli*, the BioLego predicts complete digestion of sugars by this organism, which does not happen in practice. We hypothesize that major reason for such discrepancy lays in simulation assumption of infinite growth time given to bacteria. For example (S4 Table), we measured the amount of glucose in the initial media to be 56 mg/mg DW, while after the fermentation with *E*. *coli* to be 31 mg/mg DW. In infinity, assumed by BioLego simulations, we predict complete digestion of glucose. Thus, in the setup when *S*. *cerevisiae* is grown as a second organism, it should not have any media components sufficient for ethanol production.

Another interesting hypothesis rises from the analysis of ethanol yield as a function of oxygen influx. Maximal similarity for *S*. *cerevisiae* predictions appears at low values of oxygen influx (maximum is in completely anaerobic setup), while for *E*. *coli* predictions best similarity needs at least 2mmol*gDW^-1^*h^-1^ influx of oxygen (2mmol of oxygen per 1 gram of dry cellular biomass per hour). We hypothesize that oxygen-uptake transporters in these two organisms may work with different rates (approximately 1 order of magnitude) in the same conditions. This hypothesis is well aligned with the findings of Hagman et al, who report approximate *S*. *cerevisiae* oxygen uptake rate of 3.5mmol*gDW^-1^*h^-1^ [45] and of Andersent et al, who report approximate *E*. *coli* oxygen uptake rate of 20mmol*gDW^-1^*h^-1^ [46].

## Discussion

The modular approach proposed and implemented in the BioLego Project results in notably simplifying the construction of different fermentation scenarios as well as the evaluation of different participant organisms within the scope of existing scenarios.

There are several potential directions currently considered for the next generation of BioLego. First is to provide an interface to receive models externally mapped to our envelope metabolites. Such interface will open the opportunity for evaluation of fermentation potential using additional, custom and proprietary organism models. A related direction in the abovementioned context is an integration of automatic model reconstruction algorithms, such as MIRAGE [21], as part of the BioLego framework. Another planned promising direction is to provide the interface to create more complex fermentation scenarios. Finally, we plan to incorporate knockout-optimization algorithms both from perspective of decreasing the knock-out space such as Flux Coupling Analysis[47] and from perspective of solution optimality such as OptKnock [27] and RobustKnock [28]. Currently, the BioLego project does not directly address knockout optimization, rather it provides an option to evaluate the system performance under preselected sets of knockouts of interest (including the brute-force screening of the space of single knock-out).

In parallel, we plan to work on improving the quality of BioLego predictions by adjusting the simulations to better fit wet experiments performed in parallel. Refinement of the existing organism models, integration of additional process constraints, introducing the longitudinal considerations by switching from steady-state FBA optimizations performed now to the dynamic FBA optimizations [48] are some potential approaches to this goal.

Changes in the server architecture, including support for docker infrastructures, also represent directions for potential further developments.

## Conclusion

We presented the next version of the BioLego framework, which is a freely downloadable webservice, ready for installation in the Microsoft Azure Cloud environment. BioLego provides a friendly and intuitive interface that enables the simulation (modeling and evaluation of the expected performance) and the optimization of single and two-step fermentation processes. The BioLego fermentation simulator is a scalable distributed framework, providing means to the process designers for analyzing, predicting and comparing the expected efficiency of several fermentation scenarios of interest. It is based on a novel flexible modular modelling approach, enabling smooth generation of different multi-organism fermentation configurations consisting of independent encapsulated modules, representing individual organisms.

The major contributions of this work are (1) freely downloadable BioLego Vn2.0 which is ready to install on a Microsoft Azure Cloud environment platform; and (2) Biological predictions for various fermentation scenarios supporting several published studies.

## Supporting information

S1 Fig BioLego architecture schema. (BMP)

S2 Fig All the charts resulted from reaction pairs knock-out analysis. (PDF)

S3 Fig Results for GOrilla analysis of *S*. *cerevisiae* genes, identified in knock-out analysis. (ZIP)

S1 Table Results for analysis of corn utilization. (XLSX)

S2 Table Sensitivity and gradient analyses for ethanol pro-duction using fermentation of *Kappaphycus alvarezzi* biomass. (XLSX)

S3 Table Results for analysis of two-step fermentation (*S*. *cerevisiae* followed by *E*. *coli*) of *Ulva sp*. into ethanol with knock-outs in each organis. (TXT)

S4 Table Details of experimental validation of prediction quality. (XLSX)
