# Supplementary figures and images for: Distributed flux balance analysis simulations of serial biomass fermentation by two organisms
Source: PLoS One. 2020 Jan 16;15(1):e0227363. doi: 10.1371/journal.pone.0227363 (PMC6964848; doi:10.1371/journal.pone.0227363)

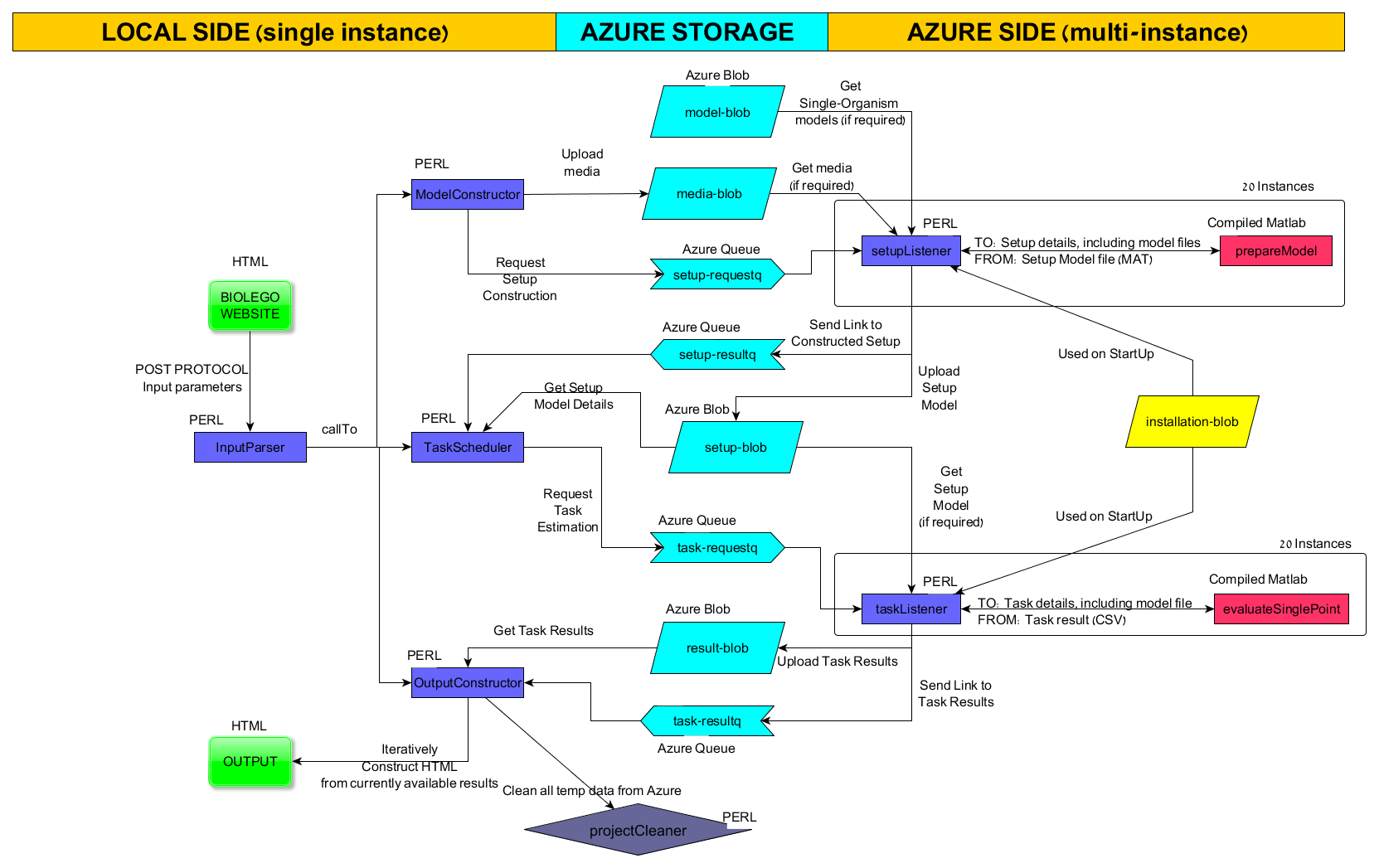

Supplement: S1 Fig — (BMP) [file pone.0227363.s001.bmp]

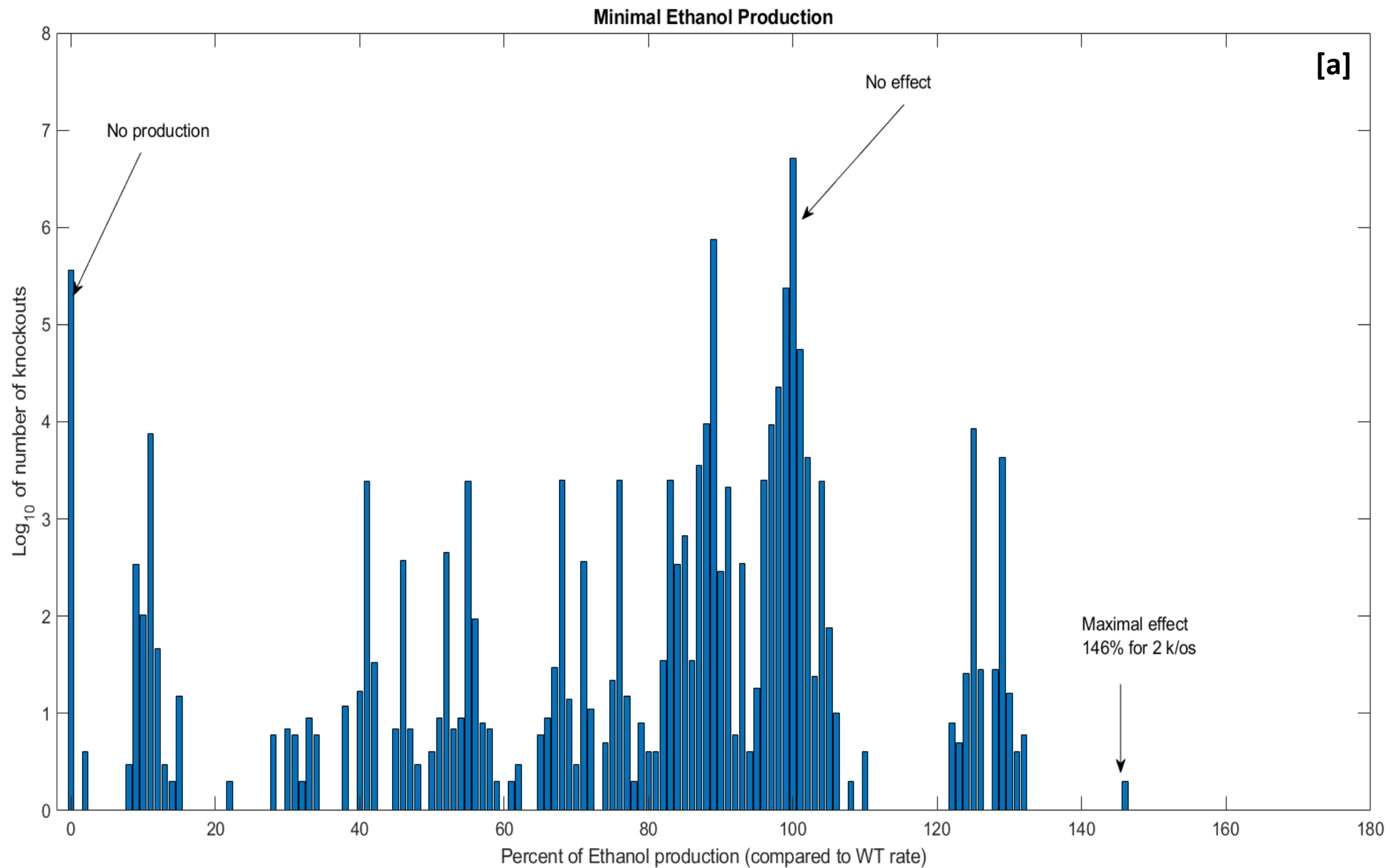

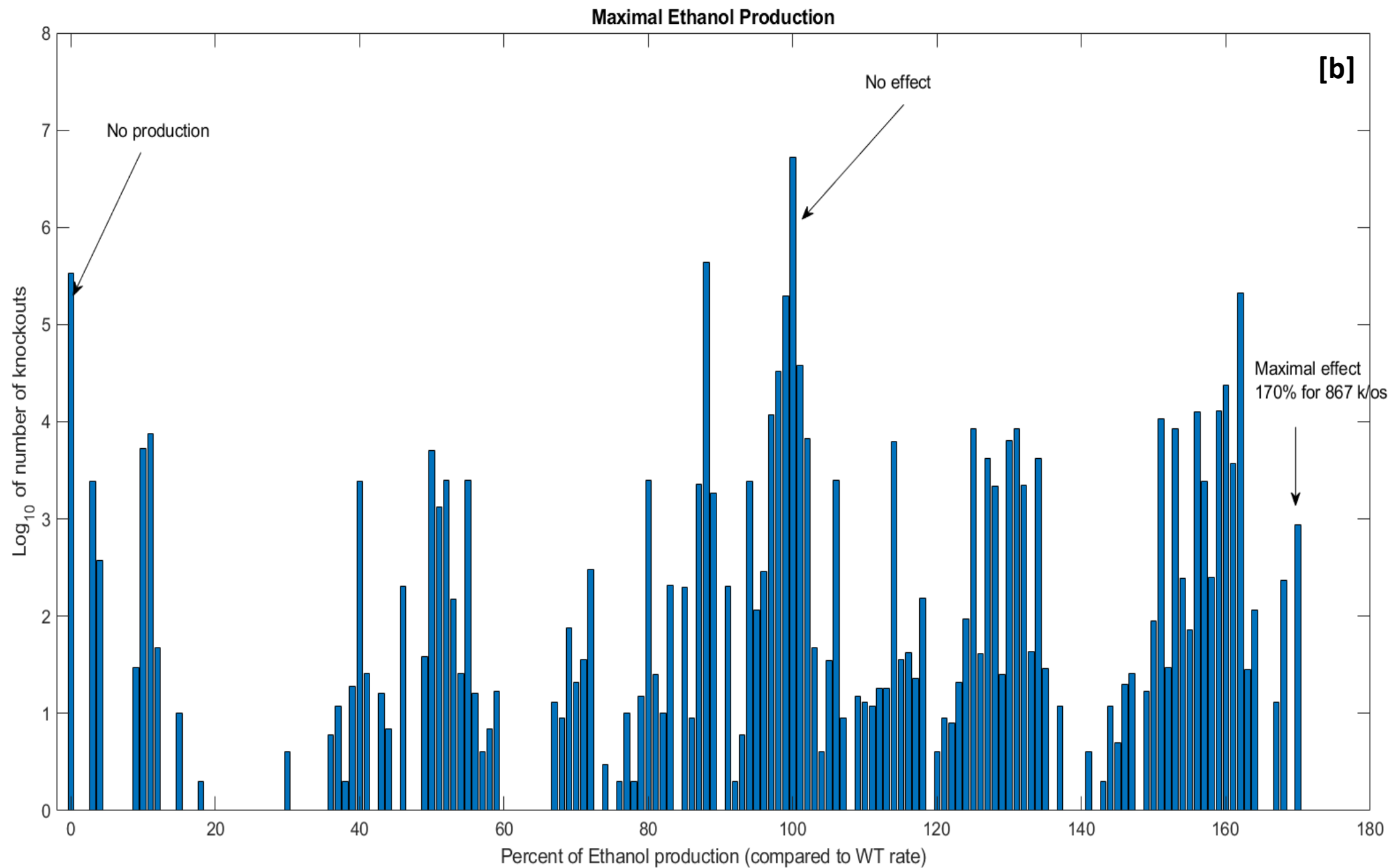

Supplement: S2 Fig — (PDF) [file pone.0227363.s002.pdf]

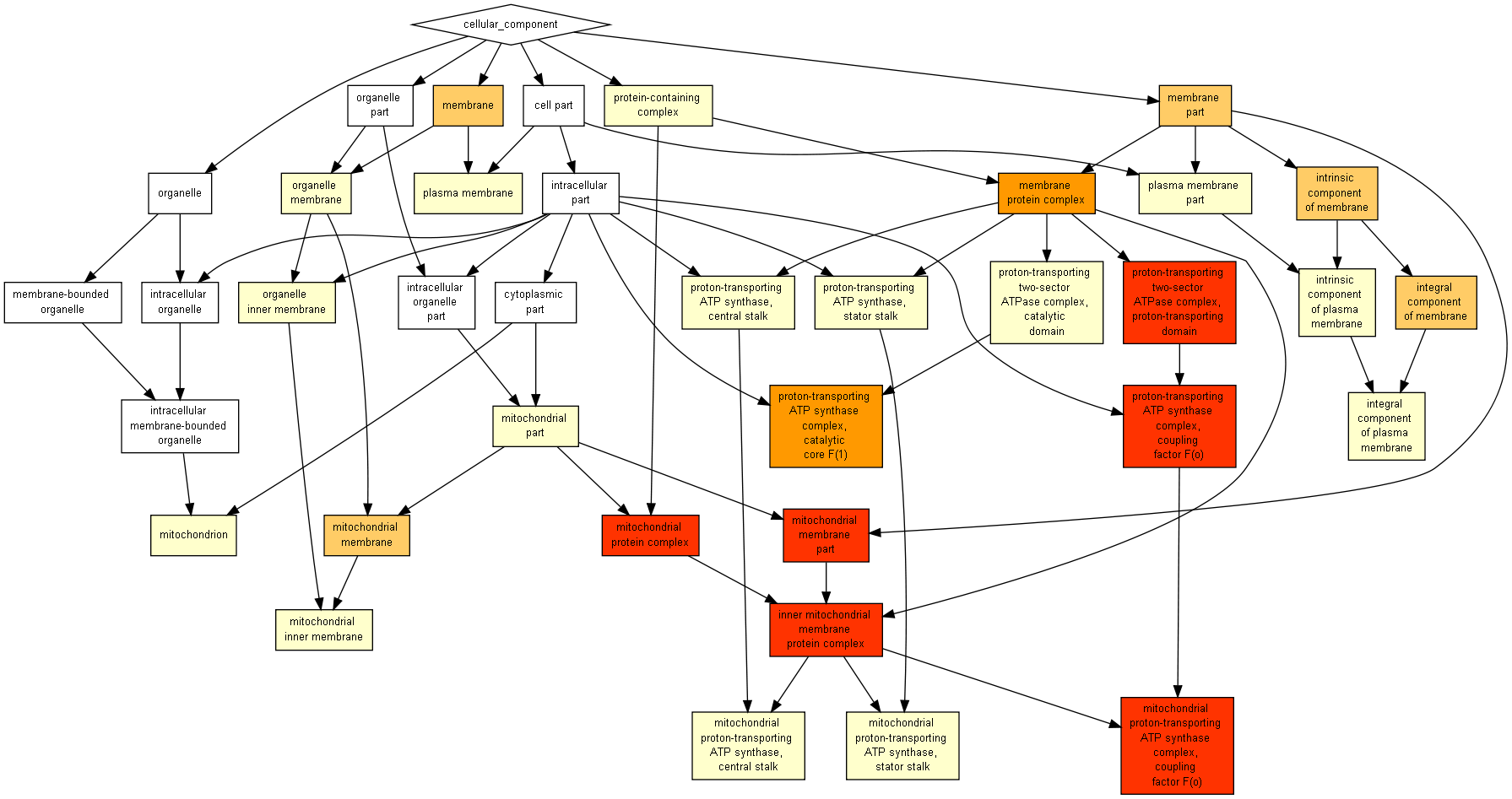

Supplement: S3 Fig — (ZIP) [file pone.0227363.s003.zip › Suppl_Figure_3.GOrilla results_files/GOResultsCOMPONENT_data/GOCOMPONENT.png]

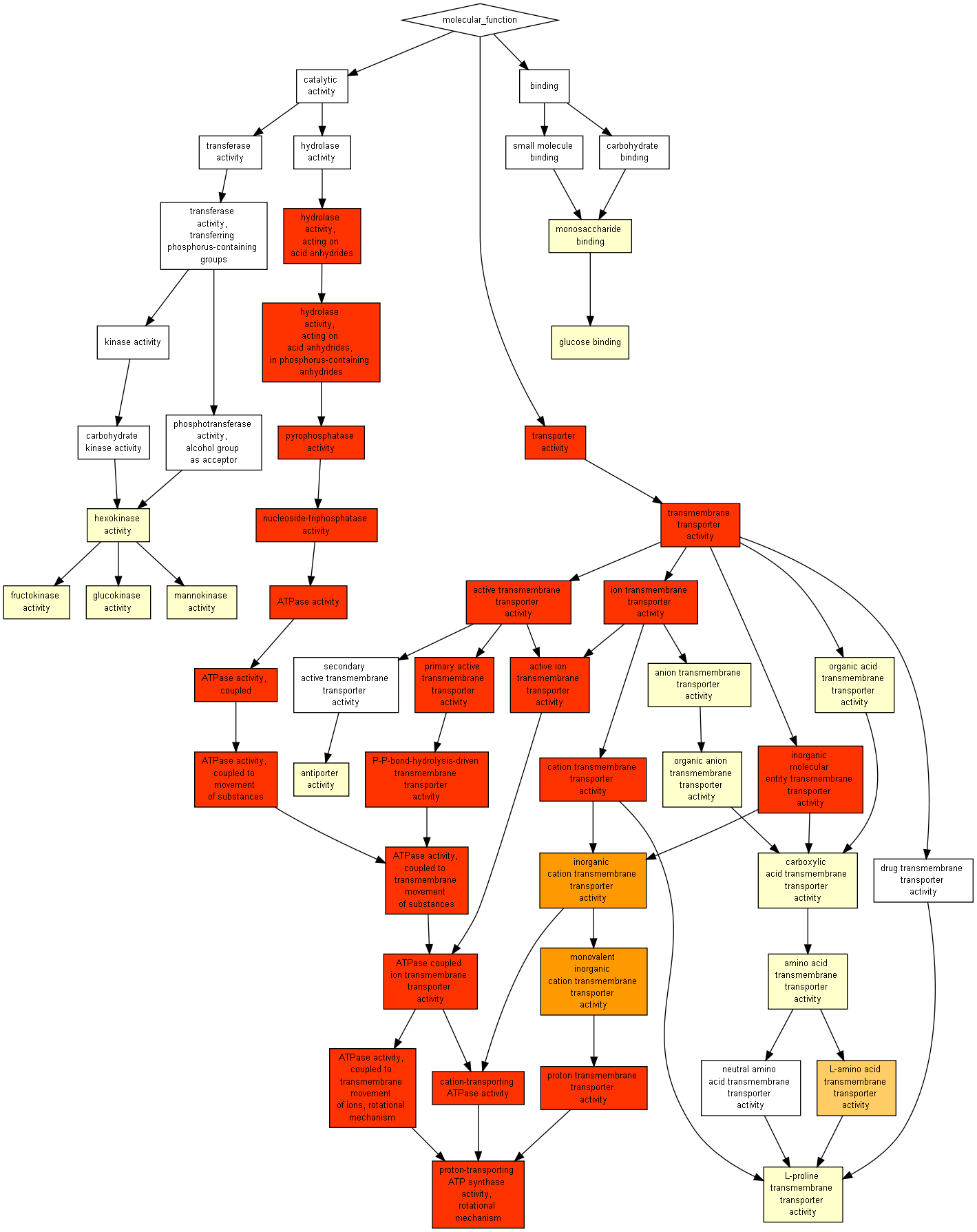

Supplement: S3 Fig — (ZIP) [file pone.0227363.s003.zip › Suppl_Figure_3.GOrilla results_files/GOResultsFUNCTION_files/GOFUNCTION.png]

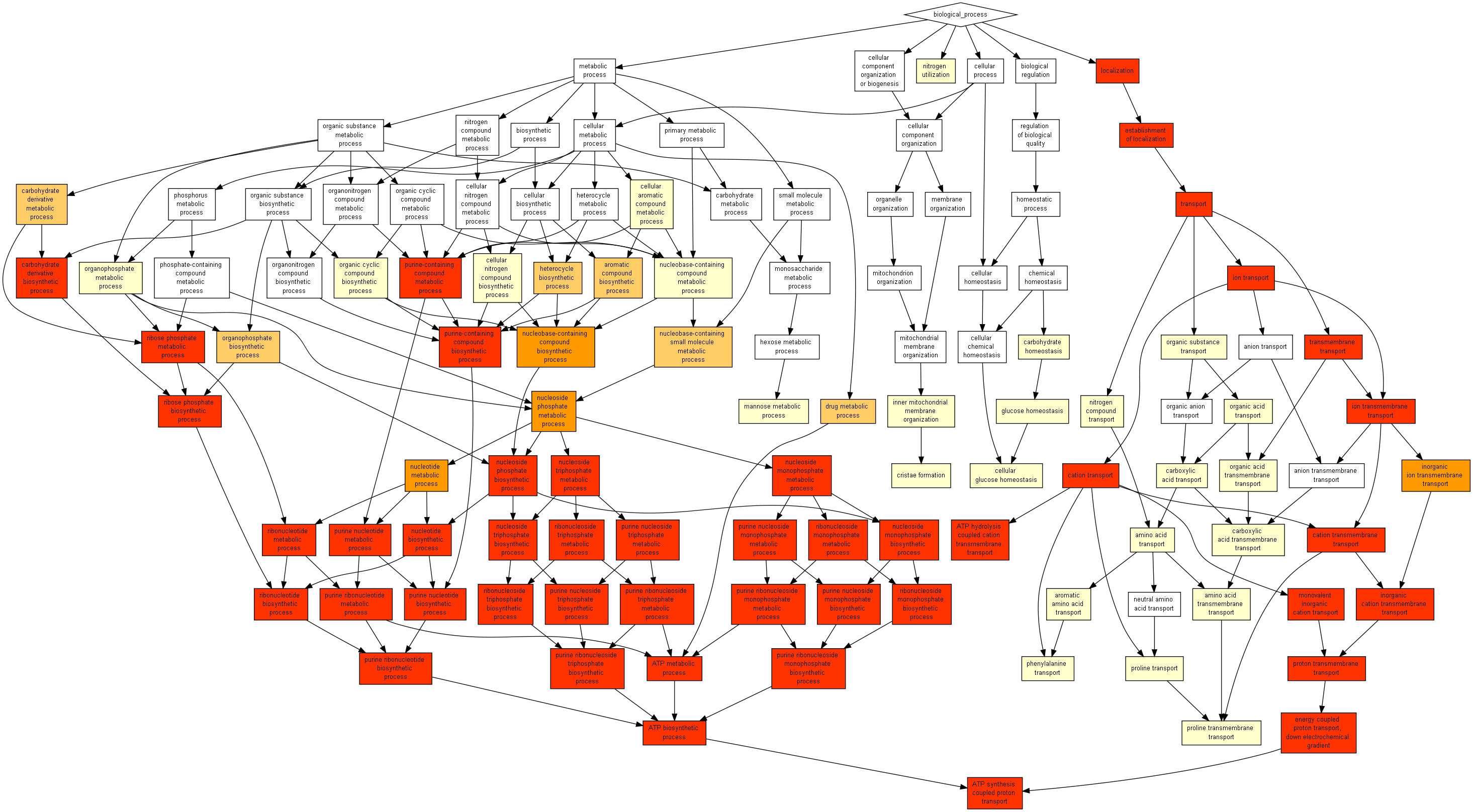

Supplement: S3 Fig — (ZIP) [file pone.0227363.s003.zip › Suppl_Figure_3.GOrilla results_files/GOResultsPROCESS_files/GOPROCESS.png]
