# Supplementary material for: Distributed flux balance analysis simulations of serial biomass fermentation by two organisms
Source: PLoS One. 2020 Jan 16;15(1):e0227363. doi: 10.1371/journal.pone.0227363 (PMC6964848; doi:10.1371/journal.pone.0227363)
Supplement: S3 Fig — (ZIP) [file pone.0227363.s003.zip › Suppl_Figure_3.GOrilla results_files/GOResultsCOMPONENT.html]

Results

**yeast kos**

*P-value color scale*

|  |  |  |  |  |
| --- | --- | --- | --- | --- |
| > 10-3 | 10-3 to 10-5 | 10-5 to 10-7 | 10-7 to 10-9 | < 10-9 |


|  |  |  |  |  |  |
| --- | --- | --- | --- | --- | --- |
| **GO term** | **Description** | **P-value** | **FDR q-value** | **Enrichment (N, B, n, b)** | **Genes** |
| GO:0000276 | mitochondrial proton-transporting ATP synthase complex, coupling factor F(o) | 1.7E-15 | 5.99E-13 | 26.14 (915,10,35,10) | [+] Show genes  ATP8 - subunit 8 of the f0 sector of mitochondrial inner membrane f1-f0 atp synthase, encoded on the mitochondrial genome; atp8 and atp6 mrnas are not translated in the absence of the f1 sector of atpase  ATP6 - mitochondrially encoded subunit a of the f0 sector of mitochondrial f1f0 atp synthase; translation is specifically activated by atp22p; atp6 and atp8 mrnas are not translated in the absence of the f1 sector of atpase  ATP20 - atp20p  ATP17 - f1f0 atp synthase subunit f  ATP7 - f1f0 atp synthase subunit d  ATP18 - atp18p  TIM11 - tim11p  ATP14 - f1f0 atp synthase subunit h  OLI1 - f0-atp synthase subunit c (atpase-associated proteolipid), encoded on the mitochondrial genome; mutation confers oligomycin resistance; expression is specifically dependent on the nuclear genes aep1 and aep2  ATP4 - atp4p |
| GO:0045263 | proton-transporting ATP synthase complex, coupling factor F(o) | 1.7E-15 | 2.99E-13 | 26.14 (915,10,35,10) | [+] Show genes  ATP8 - subunit 8 of the f0 sector of mitochondrial inner membrane f1-f0 atp synthase, encoded on the mitochondrial genome; atp8 and atp6 mrnas are not translated in the absence of the f1 sector of atpase  ATP20 - atp20p  ATP6 - mitochondrially encoded subunit a of the f0 sector of mitochondrial f1f0 atp synthase; translation is specifically activated by atp22p; atp6 and atp8 mrnas are not translated in the absence of the f1 sector of atpase  ATP7 - f1f0 atp synthase subunit d  ATP17 - f1f0 atp synthase subunit f  ATP18 - atp18p  TIM11 - tim11p  ATP14 - f1f0 atp synthase subunit h  OLI1 - f0-atp synthase subunit c (atpase-associated proteolipid), encoded on the mitochondrial genome; mutation confers oligomycin resistance; expression is specifically dependent on the nuclear genes aep1 and aep2  ATP4 - atp4p |
| GO:0098800 | inner mitochondrial membrane protein complex | 6.15E-14 | 7.21E-12 | 9.73 (915,43,35,16) | [+] Show genes  ATP8 - subunit 8 of the f0 sector of mitochondrial inner membrane f1-f0 atp synthase, encoded on the mitochondrial genome; atp8 and atp6 mrnas are not translated in the absence of the f1 sector of atpase  ATP6 - mitochondrially encoded subunit a of the f0 sector of mitochondrial f1f0 atp synthase; translation is specifically activated by atp22p; atp6 and atp8 mrnas are not translated in the absence of the f1 sector of atpase  ATP16 - f1f0 atp synthase subunit delta  ATP17 - f1f0 atp synthase subunit f  TIM11 - tim11p  ATP14 - f1f0 atp synthase subunit h  OLI1 - f0-atp synthase subunit c (atpase-associated proteolipid), encoded on the mitochondrial genome; mutation confers oligomycin resistance; expression is specifically dependent on the nuclear genes aep1 and aep2  ATP5 - atp5p  ATP15 - f1f0 atp synthase subunit epsilon  ATP20 - atp20p  ATP7 - f1f0 atp synthase subunit d  ATP1 - f1f0 atp synthase subunit alpha  ATP2 - atp2p  ATP18 - atp18p  ATP3 - atp3p  ATP4 - atp4p |
| GO:0098798 | mitochondrial protein complex | 2.8E-12 | 2.46E-10 | 7.89 (915,53,35,16) | [+] Show genes  ATP8 - subunit 8 of the f0 sector of mitochondrial inner membrane f1-f0 atp synthase, encoded on the mitochondrial genome; atp8 and atp6 mrnas are not translated in the absence of the f1 sector of atpase  ATP16 - f1f0 atp synthase subunit delta  ATP6 - mitochondrially encoded subunit a of the f0 sector of mitochondrial f1f0 atp synthase; translation is specifically activated by atp22p; atp6 and atp8 mrnas are not translated in the absence of the f1 sector of atpase  ATP17 - f1f0 atp synthase subunit f  TIM11 - tim11p  ATP14 - f1f0 atp synthase subunit h  OLI1 - f0-atp synthase subunit c (atpase-associated proteolipid), encoded on the mitochondrial genome; mutation confers oligomycin resistance; expression is specifically dependent on the nuclear genes aep1 and aep2  ATP15 - f1f0 atp synthase subunit epsilon  ATP5 - atp5p  ATP20 - atp20p  ATP7 - f1f0 atp synthase subunit d  ATP1 - f1f0 atp synthase subunit alpha  ATP2 - atp2p  ATP18 - atp18p  ATP3 - atp3p  ATP4 - atp4p |
| GO:0044455 | mitochondrial membrane part | 1E-11 | 7.06E-10 | 7.34 (915,57,35,16) | [+] Show genes  ATP8 - subunit 8 of the f0 sector of mitochondrial inner membrane f1-f0 atp synthase, encoded on the mitochondrial genome; atp8 and atp6 mrnas are not translated in the absence of the f1 sector of atpase  ATP16 - f1f0 atp synthase subunit delta  ATP6 - mitochondrially encoded subunit a of the f0 sector of mitochondrial f1f0 atp synthase; translation is specifically activated by atp22p; atp6 and atp8 mrnas are not translated in the absence of the f1 sector of atpase  ATP17 - f1f0 atp synthase subunit f  TIM11 - tim11p  ATP14 - f1f0 atp synthase subunit h  OLI1 - f0-atp synthase subunit c (atpase-associated proteolipid), encoded on the mitochondrial genome; mutation confers oligomycin resistance; expression is specifically dependent on the nuclear genes aep1 and aep2  ATP15 - f1f0 atp synthase subunit epsilon  ATP5 - atp5p  ATP20 - atp20p  ATP7 - f1f0 atp synthase subunit d  ATP1 - f1f0 atp synthase subunit alpha  ATP2 - atp2p  ATP18 - atp18p  ATP3 - atp3p  ATP4 - atp4p |
| GO:0033177 | proton-transporting two-sector ATPase complex, proton-transporting domain | 1.17E-11 | 6.86E-10 | 16.34 (915,16,35,10) | [+] Show genes  ATP8 - subunit 8 of the f0 sector of mitochondrial inner membrane f1-f0 atp synthase, encoded on the mitochondrial genome; atp8 and atp6 mrnas are not translated in the absence of the f1 sector of atpase  ATP20 - atp20p  ATP6 - mitochondrially encoded subunit a of the f0 sector of mitochondrial f1f0 atp synthase; translation is specifically activated by atp22p; atp6 and atp8 mrnas are not translated in the absence of the f1 sector of atpase  ATP7 - f1f0 atp synthase subunit d  ATP17 - f1f0 atp synthase subunit f  ATP18 - atp18p  TIM11 - tim11p  ATP14 - f1f0 atp synthase subunit h  OLI1 - f0-atp synthase subunit c (atpase-associated proteolipid), encoded on the mitochondrial genome; mutation confers oligomycin resistance; expression is specifically dependent on the nuclear genes aep1 and aep2  ATP4 - atp4p |
| GO:0098796 | membrane protein complex | 2.35E-9 | 1.18E-7 | 5.29 (915,79,35,16) | [+] Show genes  ATP8 - subunit 8 of the f0 sector of mitochondrial inner membrane f1-f0 atp synthase, encoded on the mitochondrial genome; atp8 and atp6 mrnas are not translated in the absence of the f1 sector of atpase  ATP16 - f1f0 atp synthase subunit delta  ATP6 - mitochondrially encoded subunit a of the f0 sector of mitochondrial f1f0 atp synthase; translation is specifically activated by atp22p; atp6 and atp8 mrnas are not translated in the absence of the f1 sector of atpase  ATP17 - f1f0 atp synthase subunit f  TIM11 - tim11p  ATP14 - f1f0 atp synthase subunit h  OLI1 - f0-atp synthase subunit c (atpase-associated proteolipid), encoded on the mitochondrial genome; mutation confers oligomycin resistance; expression is specifically dependent on the nuclear genes aep1 and aep2  ATP15 - f1f0 atp synthase subunit epsilon  ATP5 - atp5p  ATP20 - atp20p  ATP7 - f1f0 atp synthase subunit d  ATP1 - f1f0 atp synthase subunit alpha  ATP2 - atp2p  ATP18 - atp18p  ATP3 - atp3p  ATP4 - atp4p |
| GO:0045261 | proton-transporting ATP synthase complex, catalytic core F(1) | 6.14E-8 | 2.7E-6 | 26.14 (915,5,35,5) | [+] Show genes  ATP16 - f1f0 atp synthase subunit delta  ATP2 - atp2p  ATP1 - f1f0 atp synthase subunit alpha  ATP15 - f1f0 atp synthase subunit epsilon  ATP3 - atp3p |
| GO:0016020 | membrane | 5.82E-7 | 2.28E-5 | 1.89 (915,415,35,30) | [+] Show genes  ATP8 - subunit 8 of the f0 sector of mitochondrial inner membrane f1-f0 atp synthase, encoded on the mitochondrial genome; atp8 and atp6 mrnas are not translated in the absence of the f1 sector of atpase  ATP6 - mitochondrially encoded subunit a of the f0 sector of mitochondrial f1f0 atp synthase; translation is specifically activated by atp22p; atp6 and atp8 mrnas are not translated in the absence of the f1 sector of atpase  PUT4 - put4p  OLI1 - f0-atp synthase subunit c (atpase-associated proteolipid), encoded on the mitochondrial genome; mutation confers oligomycin resistance; expression is specifically dependent on the nuclear genes aep1 and aep2  ATP5 - atp5p  ATP20 - atp20p  TAT2 - tat2p  MEP1 - mep1p  ATP7 - f1f0 atp synthase subunit d  MEP3 - mep3p  ATP18 - atp18p  GLK1 - glucokinase  ATP3 - atp3p  MEP2 - mep2p  BAP3 - bap3p  ATP16 - f1f0 atp synthase subunit delta  DIP5 - dip5p  TPI1 - triose-phosphate isomerase tpi1  ATP17 - f1f0 atp synthase subunit f  AGP1 - agp1p  TIM11 - tim11p  ATP14 - f1f0 atp synthase subunit h  GAP1 - gap1p  ATP15 - f1f0 atp synthase subunit epsilon  ATO3 - ato3p  ATP1 - f1f0 atp synthase subunit alpha  ATP2 - atp2p  AGP3 - agp3p  BAP2 - bap2p  ATP4 - atp4p |
| GO:0044425 | membrane part | 6.34E-7 | 2.23E-5 | 2.04 (915,359,35,28) | [+] Show genes  ATP8 - subunit 8 of the f0 sector of mitochondrial inner membrane f1-f0 atp synthase, encoded on the mitochondrial genome; atp8 and atp6 mrnas are not translated in the absence of the f1 sector of atpase  ATP6 - mitochondrially encoded subunit a of the f0 sector of mitochondrial f1f0 atp synthase; translation is specifically activated by atp22p; atp6 and atp8 mrnas are not translated in the absence of the f1 sector of atpase  PUT4 - put4p  OLI1 - f0-atp synthase subunit c (atpase-associated proteolipid), encoded on the mitochondrial genome; mutation confers oligomycin resistance; expression is specifically dependent on the nuclear genes aep1 and aep2  ATP5 - atp5p  ATP20 - atp20p  MEP1 - mep1p  TAT2 - tat2p  ATP7 - f1f0 atp synthase subunit d  MEP3 - mep3p  ATP18 - atp18p  ATP3 - atp3p  BAP3 - bap3p  ATP16 - f1f0 atp synthase subunit delta  MEP2 - mep2p  DIP5 - dip5p  ATP17 - f1f0 atp synthase subunit f  AGP1 - agp1p  TIM11 - tim11p  ATP14 - f1f0 atp synthase subunit h  GAP1 - gap1p  ATP15 - f1f0 atp synthase subunit epsilon  ATO3 - ato3p  ATP1 - f1f0 atp synthase subunit alpha  ATP2 - atp2p  AGP3 - agp3p  BAP2 - bap2p  ATP4 - atp4p |
| GO:0031966 | mitochondrial membrane | 1.22E-6 | 3.91E-5 | 3.52 (915,119,35,16) | [+] Show genes  ATP8 - subunit 8 of the f0 sector of mitochondrial inner membrane f1-f0 atp synthase, encoded on the mitochondrial genome; atp8 and atp6 mrnas are not translated in the absence of the f1 sector of atpase  ATP16 - f1f0 atp synthase subunit delta  ATP6 - mitochondrially encoded subunit a of the f0 sector of mitochondrial f1f0 atp synthase; translation is specifically activated by atp22p; atp6 and atp8 mrnas are not translated in the absence of the f1 sector of atpase  ATP17 - f1f0 atp synthase subunit f  TIM11 - tim11p  ATP14 - f1f0 atp synthase subunit h  OLI1 - f0-atp synthase subunit c (atpase-associated proteolipid), encoded on the mitochondrial genome; mutation confers oligomycin resistance; expression is specifically dependent on the nuclear genes aep1 and aep2  ATP15 - f1f0 atp synthase subunit epsilon  ATP5 - atp5p  ATP20 - atp20p  ATP7 - f1f0 atp synthase subunit d  ATP1 - f1f0 atp synthase subunit alpha  ATP2 - atp2p  ATP18 - atp18p  ATP3 - atp3p  ATP4 - atp4p |
| GO:0016021 | integral component of membrane | 1.65E-6 | 4.84E-5 | 2.12 (915,321,35,26) | [+] Show genes  ATP8 - subunit 8 of the f0 sector of mitochondrial inner membrane f1-f0 atp synthase, encoded on the mitochondrial genome; atp8 and atp6 mrnas are not translated in the absence of the f1 sector of atpase  ATP6 - mitochondrially encoded subunit a of the f0 sector of mitochondrial f1f0 atp synthase; translation is specifically activated by atp22p; atp6 and atp8 mrnas are not translated in the absence of the f1 sector of atpase  PUT4 - put4p  OLI1 - f0-atp synthase subunit c (atpase-associated proteolipid), encoded on the mitochondrial genome; mutation confers oligomycin resistance; expression is specifically dependent on the nuclear genes aep1 and aep2  ATP5 - atp5p  MEP1 - mep1p  TAT2 - tat2p  ATP7 - f1f0 atp synthase subunit d  MEP3 - mep3p  ATP18 - atp18p  ATP3 - atp3p  BAP3 - bap3p  ATP16 - f1f0 atp synthase subunit delta  MEP2 - mep2p  DIP5 - dip5p  AGP1 - agp1p  TIM11 - tim11p  ATP14 - f1f0 atp synthase subunit h  GAP1 - gap1p  ATP15 - f1f0 atp synthase subunit epsilon  ATO3 - ato3p  ATP1 - f1f0 atp synthase subunit alpha  ATP2 - atp2p  AGP3 - agp3p  BAP2 - bap2p  ATP4 - atp4p |
| GO:0031224 | intrinsic component of membrane | 2.18E-6 | 5.9E-5 | 2.09 (915,325,35,26) | [+] Show genes  ATP8 - subunit 8 of the f0 sector of mitochondrial inner membrane f1-f0 atp synthase, encoded on the mitochondrial genome; atp8 and atp6 mrnas are not translated in the absence of the f1 sector of atpase  ATP6 - mitochondrially encoded subunit a of the f0 sector of mitochondrial f1f0 atp synthase; translation is specifically activated by atp22p; atp6 and atp8 mrnas are not translated in the absence of the f1 sector of atpase  PUT4 - put4p  OLI1 - f0-atp synthase subunit c (atpase-associated proteolipid), encoded on the mitochondrial genome; mutation confers oligomycin resistance; expression is specifically dependent on the nuclear genes aep1 and aep2  ATP5 - atp5p  MEP1 - mep1p  TAT2 - tat2p  ATP7 - f1f0 atp synthase subunit d  MEP3 - mep3p  ATP18 - atp18p  ATP3 - atp3p  BAP3 - bap3p  ATP16 - f1f0 atp synthase subunit delta  MEP2 - mep2p  DIP5 - dip5p  AGP1 - agp1p  TIM11 - tim11p  ATP14 - f1f0 atp synthase subunit h  GAP1 - gap1p  ATP15 - f1f0 atp synthase subunit epsilon  ATO3 - ato3p  ATP1 - f1f0 atp synthase subunit alpha  ATP2 - atp2p  AGP3 - agp3p  BAP2 - bap2p  ATP4 - atp4p |
| GO:0005743 | mitochondrial inner membrane | 3.95E-5 | 9.93E-4 | 3.56 (915,88,35,12) | [+] Show genes  ATP6 - mitochondrially encoded subunit a of the f0 sector of mitochondrial f1f0 atp synthase; translation is specifically activated by atp22p; atp6 and atp8 mrnas are not translated in the absence of the f1 sector of atpase  ATP16 - f1f0 atp synthase subunit delta  ATP7 - f1f0 atp synthase subunit d  ATP17 - f1f0 atp synthase subunit f  ATP1 - f1f0 atp synthase subunit alpha  ATP2 - atp2p  TIM11 - tim11p  ATP14 - f1f0 atp synthase subunit h  ATP3 - atp3p  ATP5 - atp5p  ATP15 - f1f0 atp synthase subunit epsilon  ATP4 - atp4p |
| GO:0019866 | organelle inner membrane | 5E-5 | 1.17E-3 | 3.49 (915,90,35,12) | [+] Show genes  ATP6 - mitochondrially encoded subunit a of the f0 sector of mitochondrial f1f0 atp synthase; translation is specifically activated by atp22p; atp6 and atp8 mrnas are not translated in the absence of the f1 sector of atpase  ATP16 - f1f0 atp synthase subunit delta  ATP7 - f1f0 atp synthase subunit d  ATP17 - f1f0 atp synthase subunit f  ATP1 - f1f0 atp synthase subunit alpha  ATP2 - atp2p  TIM11 - tim11p  ATP14 - f1f0 atp synthase subunit h  ATP3 - atp3p  ATP5 - atp5p  ATP15 - f1f0 atp synthase subunit epsilon  ATP4 - atp4p |
| GO:0000274 | mitochondrial proton-transporting ATP synthase, stator stalk | 5.14E-5 | 1.13E-3 | 26.14 (915,3,35,3) | [+] Show genes  ATP7 - f1f0 atp synthase subunit d  ATP5 - atp5p  ATP4 - atp4p |
| GO:0045265 | proton-transporting ATP synthase, stator stalk | 5.14E-5 | 1.06E-3 | 26.14 (915,3,35,3) | [+] Show genes  ATP7 - f1f0 atp synthase subunit d  ATP5 - atp5p  ATP4 - atp4p |
| GO:0045269 | proton-transporting ATP synthase, central stalk | 5.14E-5 | 1.01E-3 | 26.14 (915,3,35,3) | [+] Show genes  ATP16 - f1f0 atp synthase subunit delta  ATP15 - f1f0 atp synthase subunit epsilon  ATP3 - atp3p |
| GO:0005756 | mitochondrial proton-transporting ATP synthase, central stalk | 5.14E-5 | 9.53E-4 | 26.14 (915,3,35,3) | [+] Show genes  ATP16 - f1f0 atp synthase subunit delta  ATP3 - atp3p  ATP15 - f1f0 atp synthase subunit epsilon |
| GO:0005887 | integral component of plasma membrane | 5.19E-5 | 9.14E-4 | 3.78 (915,76,35,11) | [+] Show genes  BAP3 - bap3p  MEP2 - mep2p  TAT2 - tat2p  MEP1 - mep1p  PUT4 - put4p  DIP5 - dip5p  MEP3 - mep3p  AGP1 - agp1p  BAP2 - bap2p  AGP3 - agp3p  GAP1 - gap1p |
| GO:0031226 | intrinsic component of plasma membrane | 5.9E-5 | 9.89E-4 | 3.73 (915,77,35,11) | [+] Show genes  BAP3 - bap3p  MEP2 - mep2p  TAT2 - tat2p  MEP1 - mep1p  PUT4 - put4p  DIP5 - dip5p  MEP3 - mep3p  AGP1 - agp1p  BAP2 - bap2p  AGP3 - agp3p  GAP1 - gap1p |
| GO:0033178 | proton-transporting two-sector ATPase complex, catalytic domain | 6.32E-5 | 1.01E-3 | 10.05 (915,13,35,5) | [+] Show genes  ATP16 - f1f0 atp synthase subunit delta  ATP2 - atp2p  ATP1 - f1f0 atp synthase subunit alpha  ATP3 - atp3p  ATP15 - f1f0 atp synthase subunit epsilon |
| GO:0044459 | plasma membrane part | 1.53E-4 | 2.34E-3 | 3.38 (915,85,35,11) | [+] Show genes  BAP3 - bap3p  MEP2 - mep2p  TAT2 - tat2p  MEP1 - mep1p  PUT4 - put4p  DIP5 - dip5p  MEP3 - mep3p  AGP1 - agp1p  BAP2 - bap2p  AGP3 - agp3p  GAP1 - gap1p |
| GO:0005886 | plasma membrane | 1.91E-4 | 2.81E-3 | 2.69 (915,136,35,14) | [+] Show genes  MEP2 - mep2p  BAP3 - bap3p  PUT4 - put4p  DIP5 - dip5p  TPI1 - triose-phosphate isomerase tpi1  AGP1 - agp1p  GAP1 - gap1p  ATO3 - ato3p  MEP1 - mep1p  TAT2 - tat2p  MEP3 - mep3p  GLK1 - glucokinase  BAP2 - bap2p  AGP3 - agp3p |
| GO:0032991 | protein-containing complex | 3.03E-4 | 4.27E-3 | 2.34 (915,179,35,16) | [+] Show genes  ATP8 - subunit 8 of the f0 sector of mitochondrial inner membrane f1-f0 atp synthase, encoded on the mitochondrial genome; atp8 and atp6 mrnas are not translated in the absence of the f1 sector of atpase  ATP16 - f1f0 atp synthase subunit delta  ATP6 - mitochondrially encoded subunit a of the f0 sector of mitochondrial f1f0 atp synthase; translation is specifically activated by atp22p; atp6 and atp8 mrnas are not translated in the absence of the f1 sector of atpase  ATP17 - f1f0 atp synthase subunit f  TIM11 - tim11p  ATP14 - f1f0 atp synthase subunit h  OLI1 - f0-atp synthase subunit c (atpase-associated proteolipid), encoded on the mitochondrial genome; mutation confers oligomycin resistance; expression is specifically dependent on the nuclear genes aep1 and aep2  ATP15 - f1f0 atp synthase subunit epsilon  ATP5 - atp5p  ATP20 - atp20p  ATP7 - f1f0 atp synthase subunit d  ATP1 - f1f0 atp synthase subunit alpha  ATP2 - atp2p  ATP18 - atp18p  ATP3 - atp3p  ATP4 - atp4p |
| GO:0005739 | mitochondrion | 5.62E-4 | 7.61E-3 | 1.80 (915,320,35,22) | [+] Show genes  ATP8 - subunit 8 of the f0 sector of mitochondrial inner membrane f1-f0 atp synthase, encoded on the mitochondrial genome; atp8 and atp6 mrnas are not translated in the absence of the f1 sector of atpase  ATP6 - mitochondrially encoded subunit a of the f0 sector of mitochondrial f1f0 atp synthase; translation is specifically activated by atp22p; atp6 and atp8 mrnas are not translated in the absence of the f1 sector of atpase  ATP16 - f1f0 atp synthase subunit delta  BAP3 - bap3p  ATP17 - f1f0 atp synthase subunit f  TPI1 - triose-phosphate isomerase tpi1  TIM11 - tim11p  ATP14 - f1f0 atp synthase subunit h  OLI1 - f0-atp synthase subunit c (atpase-associated proteolipid), encoded on the mitochondrial genome; mutation confers oligomycin resistance; expression is specifically dependent on the nuclear genes aep1 and aep2  GDH2 - glutamate dehydrogenase (nad(+))  ATP5 - atp5p  ATP15 - f1f0 atp synthase subunit epsilon  ATO3 - ato3p  ATP20 - atp20p  HXK2 - hexokinase 2  ATP7 - f1f0 atp synthase subunit d  ATP1 - f1f0 atp synthase subunit alpha  ATP2 - atp2p  ATP18 - atp18p  HXK1 - hexokinase 1  ATP3 - atp3p  ATP4 - atp4p |
| GO:0031090 | organelle membrane | 6.56E-4 | 8.56E-3 | 2.11 (915,211,35,17) | [+] Show genes  ATP8 - subunit 8 of the f0 sector of mitochondrial inner membrane f1-f0 atp synthase, encoded on the mitochondrial genome; atp8 and atp6 mrnas are not translated in the absence of the f1 sector of atpase  ATP16 - f1f0 atp synthase subunit delta  ATP6 - mitochondrially encoded subunit a of the f0 sector of mitochondrial f1f0 atp synthase; translation is specifically activated by atp22p; atp6 and atp8 mrnas are not translated in the absence of the f1 sector of atpase  DIP5 - dip5p  ATP17 - f1f0 atp synthase subunit f  TIM11 - tim11p  ATP14 - f1f0 atp synthase subunit h  OLI1 - f0-atp synthase subunit c (atpase-associated proteolipid), encoded on the mitochondrial genome; mutation confers oligomycin resistance; expression is specifically dependent on the nuclear genes aep1 and aep2  ATP5 - atp5p  ATP15 - f1f0 atp synthase subunit epsilon  ATP20 - atp20p  ATP7 - f1f0 atp synthase subunit d  ATP1 - f1f0 atp synthase subunit alpha  ATP2 - atp2p  ATP18 - atp18p  ATP3 - atp3p  ATP4 - atp4p |
| GO:0044429 | mitochondrial part | 8.61E-4 | 1.08E-2 | 2.15 (915,195,35,16) | [+] Show genes  ATP8 - subunit 8 of the f0 sector of mitochondrial inner membrane f1-f0 atp synthase, encoded on the mitochondrial genome; atp8 and atp6 mrnas are not translated in the absence of the f1 sector of atpase  ATP16 - f1f0 atp synthase subunit delta  ATP6 - mitochondrially encoded subunit a of the f0 sector of mitochondrial f1f0 atp synthase; translation is specifically activated by atp22p; atp6 and atp8 mrnas are not translated in the absence of the f1 sector of atpase  ATP17 - f1f0 atp synthase subunit f  TIM11 - tim11p  ATP14 - f1f0 atp synthase subunit h  OLI1 - f0-atp synthase subunit c (atpase-associated proteolipid), encoded on the mitochondrial genome; mutation confers oligomycin resistance; expression is specifically dependent on the nuclear genes aep1 and aep2  ATP5 - atp5p  ATP15 - f1f0 atp synthase subunit epsilon  ATP20 - atp20p  ATP7 - f1f0 atp synthase subunit d  ATP1 - f1f0 atp synthase subunit alpha  ATP2 - atp2p  ATP18 - atp18p  ATP3 - atp3p  ATP4 - atp4p |

Species used: Saccharomyces cerevisiae

The system has recognized 950 genes out of 953 gene terms entered by the user.  
 8 genes were recognized by gene symbol and 942 genes by other gene IDs .  
35 duplicate genes were removed (keeping the highest ranking instance of each gene) leaving a total of 915 genes.

The GOrilla database is periodically updated using the GO database and other sources.  
The GOrilla database was last updated on Mar 17, 2018

This results page will be available on this site for one month from now (until
May 4, 2018
). You can bookmark this page and come back to it later.

  
**'P-value'** is the enrichment
p-value computed according to the mHG or HG model. This p-value is not
corrected for multiple testing of 352 GO terms.  
  
**'FDR q-value'** is the correction of the above p-value for multiple testing using the Benjamini and Hochberg (1995) method.   
Namely, for the ith term (ranked according to p-value) the FDR q-value is (p-value \* number of GO terms) / i.   
  
**Enrichment (N, B, n, b)** is defined as follows:  
N - is the total number of genes  
B - is the total number of genes associated with a specific GO term  
n - is the number of genes in the top of the user's input list or in the target set when appropriate  
b - is the number of genes in the intersection  
Enrichment = (b/n) / (B/N)  
  
**Genes:** For each GO term you can see the list of associated genes that appear in the optimal top of the list.  
Each gene name is specified by gene symbol followed by a short description of the gene   

Back to the GOrilla main page
